# Contrasts in the marine ecosystem of two Macaronesian islands: A comparison between the remote Selvagens Reserve and Madeira Island

**DOI:** 10.1371/journal.pone.0187935

**Published:** 2017-11-14

**Authors:** Alan M. Friedlander, Enric Ballesteros, Sabrina Clemente, Emanuel J. Gonçalves, Andrew Estep, Paul Rose, Enric Sala

**Affiliations:** 1 Pristine Seas, National Geographic Society, Washington, DC, United States of America; 2 Fisheries Ecology Research Lab, University of Hawai‘i at Mānoa, Honolulu, Hawai‘i, United States of America; 3 Departamento Ecología Marina, Centre d'Estudis Avançats de Blanes-CSIC, Blanes, Spain; 4 Departamento de Biología Animal, Universidad de La Laguna, Tenerife, Canary Islands, Spain; 5 MARE – Marine and Environmental Sciences Centre, ISPA – Instituto Universitário, Lisbon, Portugal; 6 Waitt Institute, La Jolla, California, United States of America; 7 Royal Geographical Society, London, United Kingdom; Universita degli Studi di Genova, ITALY

## Abstract

The islands of Madeira and Selvagens are less than 300 km apart but offer a clear contrast between a densely populated and highly developed island (Madeira), and a largely uninhabited and remote archipelago (Selvagens) within Macaronesia in the eastern Atlantic. The Madeira Archipelago has ~260,000 inhabitants and receives over six million visitor days annually. The Selvagens Islands Reserve is one of the oldest nature reserves in Portugal and comprises two islands and several islets, including the surrounding shelf to a depth of 200 m. Only reserve rangers and a small unit of the maritime police inhabit these islands. The benthic community around Selvagens was dominated by erect and turf algae, while the community at Madeira was comprised of crustose coralline and turf algae, sessile invertebrates, and sea urchin barrens. The sea urchin *Diadema africanum* was 65% more abundant at Madeira than at Selvagens. Total fish biomass was 3.2 times larger at Selvagens than at Madeira, and biomass of top predators was more than 10 times larger at Selvagens. Several commercially important species (e.g., groupers, jacks), which have been overfished throughout the region, were more common and of larger size at Selvagens than at Madeira. Important sea urchin predators (e.g., hogfishes, triggerfishes) were also in higher abundance at Selvagens compared to Madeira. The effects of fishing and other anthropogenic influences are evident around Madeira. This is in stark contrast to Selvagens, which harbors healthy benthic communities with diverse algal assemblages and high fish biomass, including an abundance of large commercially important species. The clear differences between these two island groups highlights the importance of expanding and strengthening the protection around Selvagens, which harbors one of the last intact marine ecosystems in the North Atlantic, and the need to increase management and protection around Madeira.

## Introduction

Macaronesia is a collection of four archipelagos (Madeira, Selvagens, Azores, and Canaries) located in the North Atlantic Ocean off the coasts of Europe and Africa [1–3], although Cape Verde is also included when considering terrestrial ecosystems [4]. The Macaronesian biogeographic region has long been noted for its terrestrial biodiversity and high endemism [5]. It also has a unique marine fauna, which has been influenced by west Africa, the Mediterranean Sea, and continental western Europe [1–3, 6–7].

**Fig 1:**
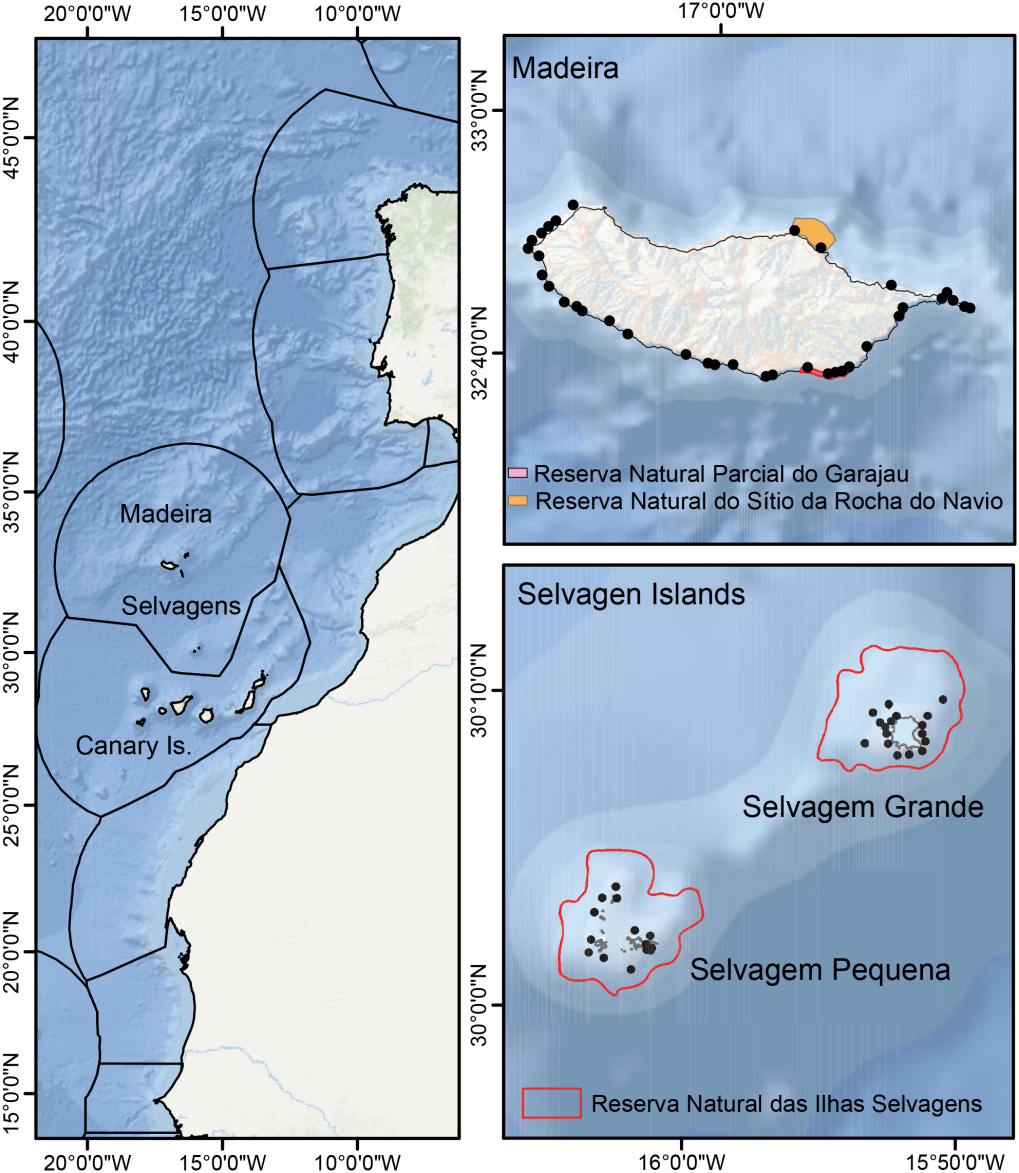
Sampling locations around Madeira and Selvagens in the eastern Atlantic Ocean.

Madeira is a Portuguese archipelago within Macaronesia and is situated ~ 650 km west of Morocco at its closest point (Fig 1). The archipelago includes the islands of Madeira (pop. 262,456), Porto Santo (pop. 5,483), and the Desertas, which consists of three uninhabited islands: Deserta Grande, Bugio and Ilhéu de Chão. The island of Madeira is a large shield volcano that rises > 6,000 m and is the largest island of the group (741 km^2^, [8]). It is home to Funchal, which is the capital and principal city of the Autonomous Region of Madeira. Tourism is the most important sector of the economy of the archipelago, with > 6.5 million overnight stays reported in 2014 [9].

The Selvagens Islands are located ~ 290 km southeast of Madeira and 170 km north of Tenerife, Canary Islands. They consist of two major islands (Selvagem Grande and Selvagem Pequena), and several small islets of varying sizes. The islands are administered by the Portuguese municipality of Funchal, and are part of the Madeira civil parish of Sé. In 1971, the Portuguese government acquired these islands, converting them into a strict nature reserve [10]. The Selvagens Islands Nature Reserve includes the surrounding shelf to a depth of 200 m (total marine area—34 km^2^) and is one of the oldest nature reserves in Portugal [10]. On Selvagem Grande, there is a permanent station with two year-around rangers and two members of the maritime police, while Selvagem Pequena is typically manned by two rangers between May and October. These are the only human inhabitants on these islands. The Selvagens Islands Nature Reserve is listed as an IUCN Protected Area Category Ia. Human activity is strictly controlled, with visits on land restricted to paths and a small area at the administrative facility. The average number of permits per year to visit the reserve is 122, involving ~ 550 people. The Selvagens are protected under European Commission legislation, included within the Natura 2000 Network as a Special Conservation Area, and designated a Special Protection Area (SPA) under the Birds Directive of the European Union [11]. It is the world’s largest breeding area for Cory’s Shearwaters (*Calonectris diomedea*, [12]), and is important to a number of other endangered and threatened sea bird species that are known to breed there [13].

Since its discovery and colonization in the 15^th^ century, Madeira has had a long history of fishing [14]. The narrow and steep insular platforms, along with low productivity waters, limit the available habitat for demersal fisheries species, and restricts the fishing methods that are suitable for these habitats [15]. Intensive overfishing of many prized nearshore species has been a concern throughout the region for decades [16–18]. This has not only reduced nearshore fish stocks, but has also had a cascading effect on the entire ecosystem by removing the predators of sea urchins, which have proliferated in the absence of these predators. This hyper-abundance of sea urchins has subsequently grazed down the erect vegetative framework of the reefs, creating barrens that have further reduced the productivity of nearshore ecosystems [19–21].

In contrast to most locations in the region, the Selvagens Islands are remote and nearly uninhabited, allowing for comparisons of marine ecosystems between two distinct levels of anthropogenic influence, while still within the same biogeographic region. Because of its remoteness and strict protection, the Selvagens Islands may represent one of the last remaining intact marine ecosystems in the eastern Atlantic. With this in mind, we set out to examine the value of Selvagens as a baseline for ecosystem health that can be used to assess ecosystem state not only within the region, but also globally. Our main objective was to compare the benthic communities and fish assemblages around a densely populated and highly developed island (Madeira) with the Selvagens Islands Nature Reserve, a strict marine protected area with almost no direct human impacts. An additional goal of our research was to examine the efficacy of marine protected areas around Madeira, comparing them to the Selvagens Islands Nature Reserve, and fished areas around Madeira.

## Materials and methods

### Ethics statement

Data were collected by all authors in a collaborative effort. Non-invasive research was conducted, which included photographs and visual estimates described in the methods. The Serviço do Parque Natural da Madeira granted all necessary permission to conduct this research. No vertebrate sampling was conducted and therefore no approval was required by the Institutional Animal Care and Use Committee. Our data are available at Data Dryad: doi:10.5061/dryad.322q2.

### Sampling design

We conducted *in situ* surveys of fishes, algae, and benthic macro-invertebrates within two depth strata (10 and 20 m) at 29 locations at Selvagens in September 2015 and 36 locations around Madeira in July 2016 (Fig 1). Oceanographic conditions (e.g., sea surface temperature, Chlorophyll-*a*) are similar around both locations during the time periods when sampling was conducted [22], thus reducing the effects of seasonality. Previous studies in the region have not shown seasonal differences in reef fish abundance [16, 23], and we therefore feel that our sampling effort in not confounded by potential temporal variability in these reef fish assemblages. Sampling locations were restricted to rocky boulder habitat at both locations to reduce the influence of habitat on fish assemblage structure, which is known to exist around Madeira [24]. Samples were allocated haphazardly between the 10 and 20 m bathymetric contour, with sites spaced ~ 1 km apart around Selvagens and ~ 2.5 km apart around Madeira. Weather conditions precluded sampling along parts of the north coast of Madeira.

At Madeira Island, the Garajau Marine Protected Area (MPA) prohibits the take of marine life except bait fish for the tuna fishery, which consists of small coastal pelagic species [e.g., mainly Atlantic chub mackerel (*Scomber colias*), European pilchard (*Sardina pilchardus*) and bogue (*Boops boops*)]. The Rocha do Navio MPA permits extraction of nearshore resources using spearfishing and line fishing, as well as collecting of invertebrates and tuna bait fishing. Samples from the Garajau MPA were excluded in overall comparisons between Madeira and Selvagens as it was the only MPA with a high level of protection at Madeira. The Garajau MPA accounted for only 4.4% of the coastline around Madeira and its exclusion allowed for a better comparison of the two island groups without the confounding effect of this small (3.76 km^2^) MPA.

All surveys were conducted on open-circuit scuba between the hours of 9:00 and 16:00 to reduce the influence of crepuscular variation in the fish assemblages. Dive duration ranged from 60–90 min depending on habitat and environmental conditions.

### Benthos

Characterization of the benthos was conducted along one 50 m-long transect run parallel to the shoreline at each of the two depth strata. For algae and sessile invertebrates, we used a line-point intercept methodology along each transect, recording the species or taxa found every 20 cm on a fiberglass measuring tape. Sessile benthic cover was grouped into turf algae (< 3 cm in height, e.g., *Caulerpa webbiana*, *Cottoniella filamentosa*), erect algae (e.g., *Halopteris scoparia*, *Padina pavonica*), canopy algae (e.g., *Cystoseira abies-marina*, *Sargassum* spp.), erect non-canopy algae (e.g., *Lobophora variegata*), crustose coralline algae (CCA, e.g., *Neogoniolithon* spp.), sessile invertebrates (e.g., *Balanus* sp.), and barrens, which consisted of bare rock. Sponges accounted for <5% of the sessile cover at both island groups and were included as sessile invertebrates. For mobile invertebrates, we counted individuals in twenty-five 50 x 50 cm quadrats randomly placed along each of the 50-m transects.

### Fishes

At each depth stratum within a site, divers counted and estimated lengths of all fishes encountered within fixed-length (25-m) belt transects whose widths differed depending on the direction of swim. All fish ≥ 20 cm total length (TL) were tallied within a 4-m wide strip surveyed on an initial “swim-out” as the transect line was laid out (transect area = 100 m^2^). All fishes < 20 cm TL were tallied within a 2-m wide strip surveyed on the return swim back along the laid transect line (transect area = 50 m^2^). Swimming duration per transect varied from 10–15 min, depending on habitat complexity and fish abundance. Three replicate transects were performed per site at each depth stratum.

Fishes were identified to species level in all cases. Fish total length (TL) was estimated to the nearest cm. Fishes were tallied by length and individual-specific lengths were converted to body weights. Numerical density (abundance) was expressed as number of individuals per m^2^ and biomass density was expressed as g per m^2^. The biomass of individual fishes was estimated using the allometric length-weight conversion: W = aTL^b^, where parameters a and b are species-specific constants, TL is total length in cm, and W is weight in grams. Length-weight fitting parameters were obtained from FishBase [25]. The sum of all individual weights and numerical densities was used to estimate biomass and numerical density by species. Fish species diversity were calculated from the Shannon-Weaver diversity index: $\sum_{i\;=\;1}^{R}\left(p\text{i}\;\text{X}\;[lnp\text{i}\right])$, where *p*_*i*_ is the proportion of all individuals counted that were of taxa *i*. Fishes were categorized into four trophic groups (top predators, herbivores, secondary consumers, and planktivores) based on information from FishBase [25]. Resource (commercially targeted) species were designated based on expert opinion of dive operators, fishers, and resource managers.

### Statistical analysis

Principal Coordinate Analysis (PCO) was used to compare sessile benthic functional groups between islands. Data were arcsine square root transformed prior to analysis. Drivers of sessile benthic community structure were investigated using permutation-based multivariate analysis of variance (PERMANOVA). Similarity percentages analysis (SIMPER) was used to examine differences in sessile benthic functional group cover between islands and depths. PCO, SIMPER, and PERMANOVA were also used to compare mobile invertebrates and fishes between island groups. Densities (individuals m^-2^) of the top 10 mobile invertebrate species, which together accounted for > 90% of the individuals in each island group, were square root transformed prior to all analyses. Fish assemblage structure by biomass (g m^-2^) was square root transformed, while numerical abundance (individuals m^-2^) was ln(x+1) transformed prior to multivariate analyses. All PERMANOVA, PCOs, and SIMPER analyses were conducted using Primer v6 [26].

Fish assemblage characteristics and trophic biomass between islands were compared using linear mixed models (LMMs) with island group, depth strata, and their interaction treated as fixed factors. Stations were treated as a random effect to account for spatial autocorrelation in these data. Unplanned comparisons between pairs were examined using the Tukey-Kramer HSD (honestly significant difference) test (α = 0.05). Numerical abundance, total biomass, resource fish biomass, and trophic group biomass were all ln(x+1) transformed prior to conducting the LMMs.

Fish trophic group biomass was tested for differences between islands and depths using multivariate analysis of variance (MANOVA). The multivariate test statistic Pillai’s Trace was used because it is robust to heterogeneity of variance and is less likely to involve type I errors than are comparable tests [27]. Canonical discriminate analysis was used to identify and display the nature of the significant differences among islands and depths found by the MANOVA. Trends in the trophic groups were represented as vectors given by correlations of these variables with the canonical variates. These vectors were plotted on the first two canonical axes, together with the treatment centroids and 95% confidence clouds. The strength of each variable in discriminating among groups was displayed graphically as the length of these vectors. Trophic group biomasses were ln(x+1) transformed to conform to the assumption of multivariate normality prior to analysis. We performed univariate GLMs, as described above, if the MANOVA was significant.

Although our main focus was on comparisons between Selvagens and Madeira, the few small MPAs around Madeira provided insights into the potential benefits of protection within this highly developed island. Owing to small sample sizes, comparisons of total fish biomass among MPAs on Madeira with Selvagens and fished areas around Madeira were conducted using a Kruskal-Wallis rank-sum test, with Dunn’s test for unplanned multiple comparisons [28]. All LMM, MANOVA and non-parametric analyses were performed using JMP Pro 12.2 [29].

Overall community structure between islands and depths were compared using Principal Components Analysis on dominant sessile benthic cover, mobile invertebrates, and fish biomass by trophic group with supplemental variables (island, depth strata) projected onto the unconstrained ordination using the ordination program CANOCO version 5.0 [30]. Sessile benthic cover data were arcsine square root transformed, mobile invertebrates and fish biomass were ln(x+1) transformed prior to analysis. Observations in the input matrix were unique sampling stations (n = 122). Functional groups were centered (subtraction of the average of the column values) and standardized (division by the standard deviation of the column values), resulting in each column having zero mean and unit variance.

## Results

### Benthic community

There was a significant difference in the assemblages of sessile benthic functional groups between islands (Pseudo-F_1,121_ = 35.6, p < 0.001) and between depths (Pseudo-F_1,121_ = 3.6, p = 0.02), but not in their interaction (Pseudo-F_1,121_ = 0.1, p = 0.91). Turf algae cover (35%) and erect algae (30%) accounted for the majority of the sessile benthic cover around Selvagens, while sessile cover at Madeira was dominated by crustose coralline algae (CCA, 30%), turf algae (21%), sessile invertebrates (16%, primarily *Balanus* sp.), erect algae (12%), and barrens (11%) (Table 1). Average dissimilarity in sessile benthic functional group cover between islands was 58.6%, with turf algae contributing the most to this dissimilarity (22%), followed by CCA (22%), and erect algae (20%) (Table 1). Average dissimilarity of sessile benthic cover between depths was 47.8% (Table 1). Turf algal cover was higher at 20 m and contributed 24% to this dissimilarity, followed by CCA (18%), which was also higher at 20 m. Erect algae was higher at 10 m and contributed an additional 15% to the dissimilarity between islands.

**Table 1:** A. Similarity of Percentages (SIMPER) for sessile benthic cover by functional group most responsible for the percent dissimilarities between islands using Bray-Curtis similarity analysis of hierarchical agglomerative group average clustering. Average dissimilarity = 58.6%, with one standard deviation of the mean in parentheses. B. SIMPER for sessile benthic cover by functional group most responsible for the percent dissimilarities between depths. Average dissimilarity = 47.8%. Abundance values are means and one standard deviation of the mean in parentheses.

A	Madeira	Selvagens	Avg. Diss.	% contrib.	Cum. %
Turf	21.4 (22.8)	34.6 (18.7)	13.1 (1.5)	22.4	22.4
CCA	30.1 (22.8)	5.9 (5.8)	13.0 (1.2)	22.3	44.6
Erect algae	9.2 (15.8)	29.7 (14.4)	11.5 (1.5)	19.7	64.3
Inverts	15.9 (16.8)	6.1 (6.4)	7.0 (1.0)	12.0	76.3
Encrusting algae	11.8 (15.1)	10.2 (9.5)	6.1 (0.9)	10.4	86.6
Barren	11.0 (10.1)	8.4 (10.5)	5.1 (1.0)	8.7	95.4
B	10 m	20 m	Avg. Diss.	% contrib.	Cum. %
Turf	24.8 (18.4)	30.5 (24.7)	11.7 (1.3)	24.5	24.5
CCA	19.1 (21.3)	18.1 (20.5)	8.4 (1.0)	17.6	42.0
Erect algae	22.0 (15.7)	15.9 (17.5)	7.4 (1.2)	15.5	57.6
Encrusting algae	13.7 (11.0)	8.4 (14.7)	6.9 (1.1)	14.5	72.1
Inverts.	9.7 (13.4)	12.7 (14.1)	6.4 (0.9)	13.3	85.4
Barren	8.2 (8.2)	11.3 (11.3)	5.0 (1.0)	10.5	95.9

**Fig 2:**
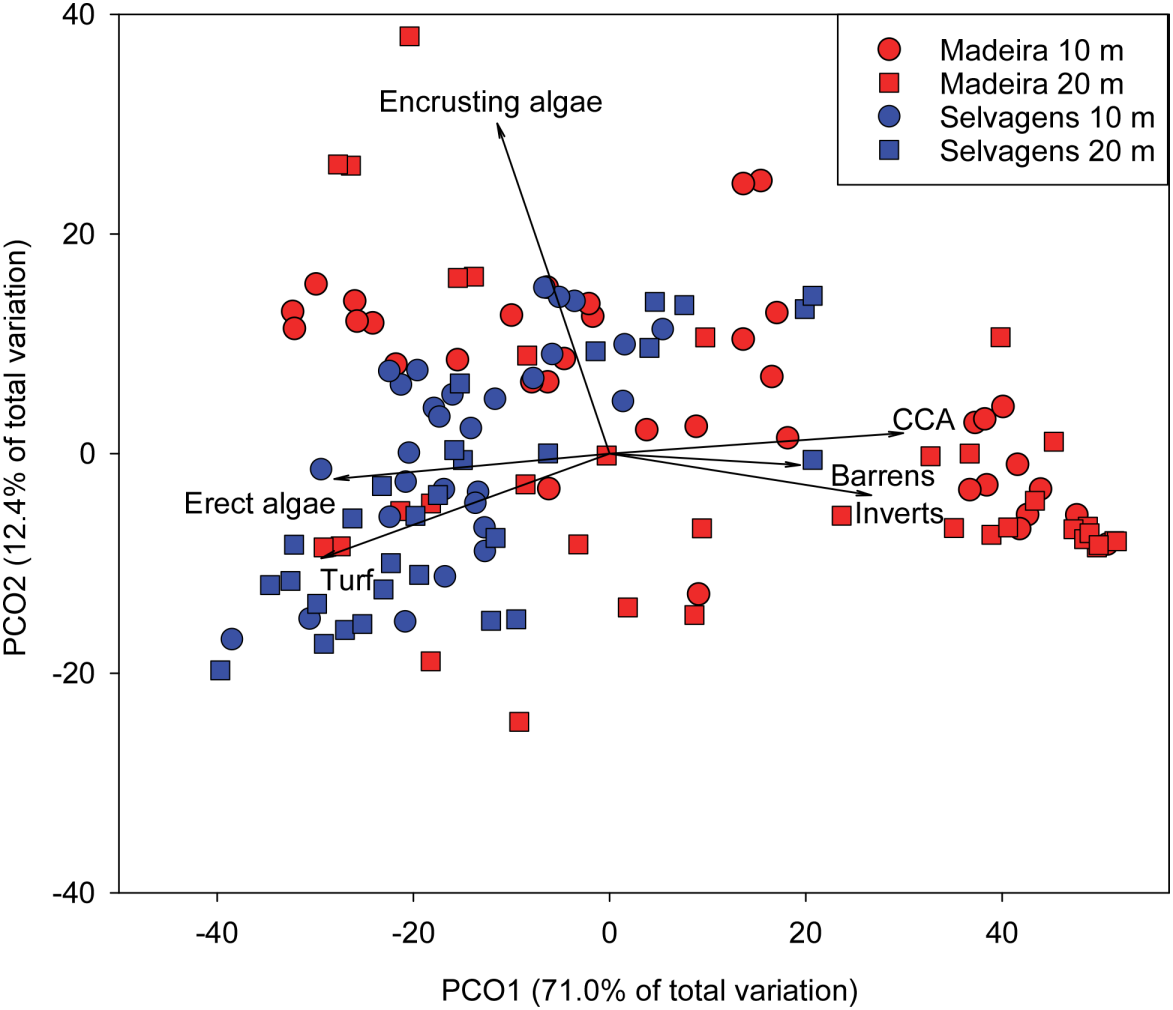
Principal Coordinate Analysis of sessile benthic functional groups. Percent cover data were arcsine square root transformed prior to analysis.

Analysis of sessile benthic functional assemblage structure showed clear separation in ordination space, with sites at Selvagens showing higher concordance relative to Madeira (Fig 2). PCO1 explained 71% of the variation in sessile benthic functional cover between islands, with CCA, barrens, and invertebrates trending in the direction of Madeira and erect algae and turf in the direction of Selvagens. PCO2 only explained 12% of the variation in sessile benthic functional cover, with encrusting algae trending towards shallower sites.

### Mobile invertebrates

There was a significant difference in the assemblages of mobile benthic functional groups between islands (Pseudo-F_1,121_ = 22.5, p < 0.001) and between depths (Pseudo-F_1,121_ = 14.2, p < 0.001) but not in their interaction (Pseudo-F_1,121_ = 0.5, p = 0.79). The sea urchin *Diadema africanum* was the most common mobile invertebrate observed at both islands (Table 2). The average density at Madeira (4.4 individuals m^-2^, ± 4.4 sd) was however 65% higher when compared with Selvagens (2.7 individuals m^-2^, ± 2.7 sd), and accounted for 45.5% of the dissimilarity between island groups. The hermit crab, *Calcinus tubularis*, was the second most abundant mobile invertebrate at Madeira, while at Selvagens the purple sea urchin, *Paracentrotus lividus* was most abundant. Densities of *D*. *africanum* were 35% higher at the 20 m sites compared with the 10 m sites and accounted for 45.0% of the dissimilarity between depths (Table 2). In contrast, densities of the black (*Arbacia lixula*) and purple (*P*. *lividus*) sea urchins were > 4 times higher at 10 m compared with 20 m, although these species were much more abundant at Selvagens compared with Madeira.

**Table 2:** A. SIMPER for mobile invertebrates most responsible for the percent dissimilarities between islands using Bray-Curtis similarity analysis of hierarchical agglomerative group average clustering. Average dissimilarity = 71.5%, with one standard deviation of the mean in parentheses. B. SIMPER for mobile invertebrates most responsible for the percent dissimilarities between depths. Average dissimilarity = 66.8%. Abundance values are means and one standard deviation of the mean in parentheses.

**A**	**Species**	**Madeira**	**Selvagens**	**Avg Diss**.	**% contrib**.	**Cum. %**
**Diadematoida**	*Diadema africanum*	4.42 (4.41)	2.70 (2.77)	32.6 (1.4)	45.5	45.5
**Decapoda**	*Calcinus tubularis*	1.12 (1.85)	0.01 (0.03)	10.1 (0.8)	14.1	59.6
**Camarodonta**	*Paracentrotus lividus*	0.06 (0.31)	1.31 (3.27)	8.4 (0.5)	11.8	71.3
**Arbacioida**	*Arbacia lixula*	0.45 (1.43)	0.81 (1.24)	6.5 (0.7)	9.1	80.4
**Decapoda**	*Percnon gibbesi*	0.32 (0.40)	0.14 (0.23)	3.5 (0.7)	4.9	85.3
**Amphinomida**	*Hermodice carunculata*	0.32 (0.51)	0.16 (0.21)	3.0 (0.7)	4.2	89.5
**Sessilia**	*Megabalanus azoricus*	-	0.34 (1.24)	2.6 (0.3)	3.7	93.2
**B**	Species	10 m	20 m	Avg Diss.	% contrib.	Cum. %
**Diadematoida**	*Diadema africanum*	3.06 (3.69)	4.14 (3.88)	30.1 (1.3)	45.0	45.0
**Arbacioida**	*Arbacia lixula*	1.00 (1.69)	0.23 (0.70)	8.5 (0.8)	12.7	57.7
**Camarodonta**	*Paracentrotus lividus*	1.06 (3.04)	0.25 (1.22)	8.2 (0.5)	12.3	69.9
**Decapoda**	*Calcinus tubularis*	0.78 (1.67)	0.40 (1.17)	6.2 (0.6)	9.3	79.2
**Decapoda**	*Percnon gibbesi*	0.32 (0.40)	0.15 (0.26)	3.3 (0.8)	4.7	84.1
**Amphinomida**	*Hermodice carunculata*	0.16 (0.38)	0.34 (0.42)	3.0 (0.8)	4.5	88.5
**Sessilia**	*Megabalanus azoricus*	0.30 (1.21)	0.02 (0.16)	2.8 (0.3)	4.1	92.7

**Fig 3:**
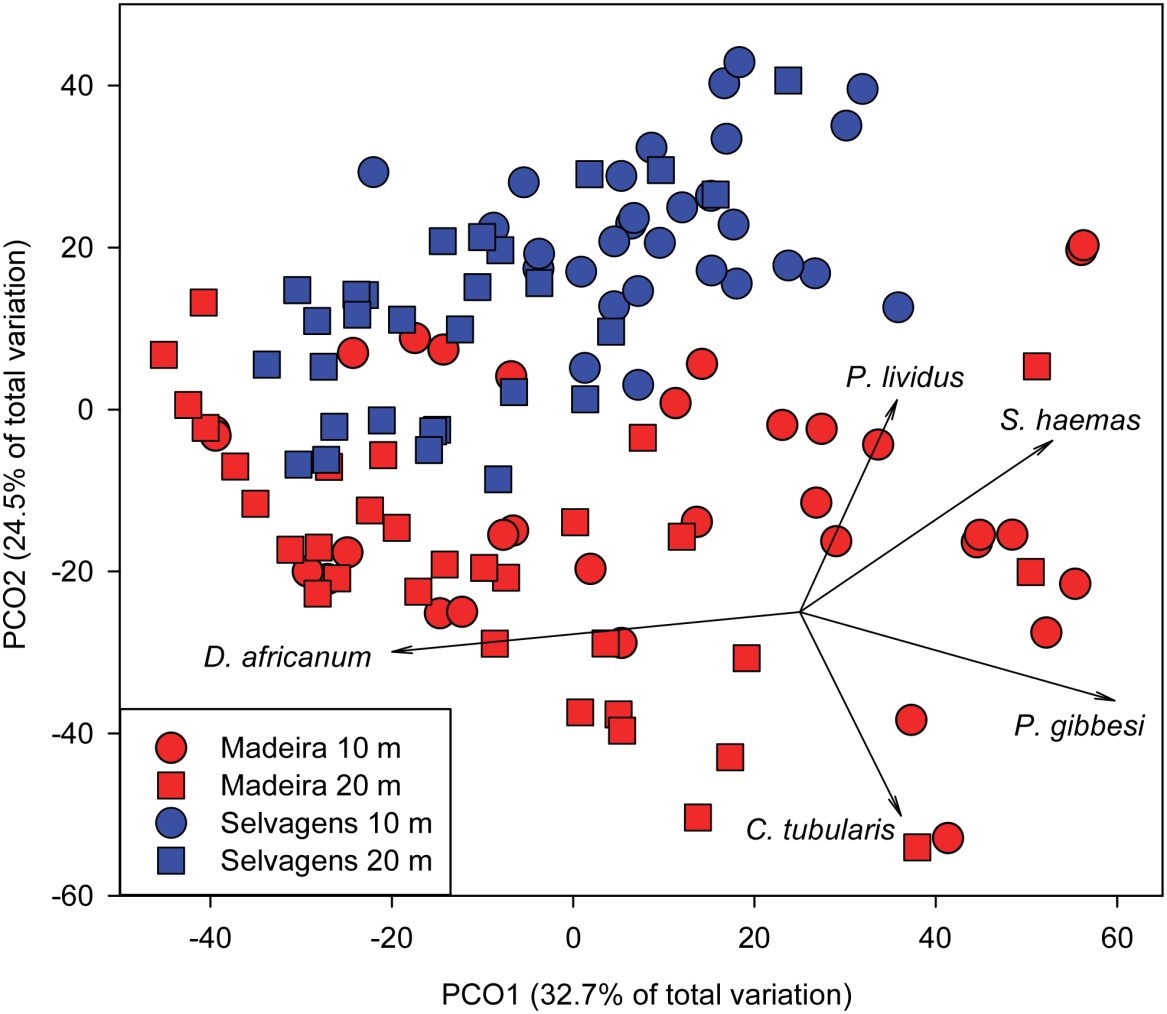
Principal Coordinate Analysis of mobile invertebrate communities between islands and depths. Top 10 species together accounted for > 90% of the individuals in each island group. Data square root transformed prior to analysis.

Analysis of mobile invertebrate densities showed clear separation in ordination space between islands, with Selvagens showing higher concordance among sites (Fig 3). PCO1 explained 32.7% of the variation in mobile invertebrate assemblage structure and was correlated with the combination of island and depth. PCO2 explained an additional 24.5% of the variation. *D*. *africanum*, along with *Stramonita haemastoma* and *Percnon gibbesi* drove much of the separation along PCO1, while *P*. *lividus* and *C*. *tubularis* were orthogonal to these species, with the former explaining variation at Selvagens and the latter at Madeira.

### Fishes

#### Fish assemblage characteristics

**Fig 4:**
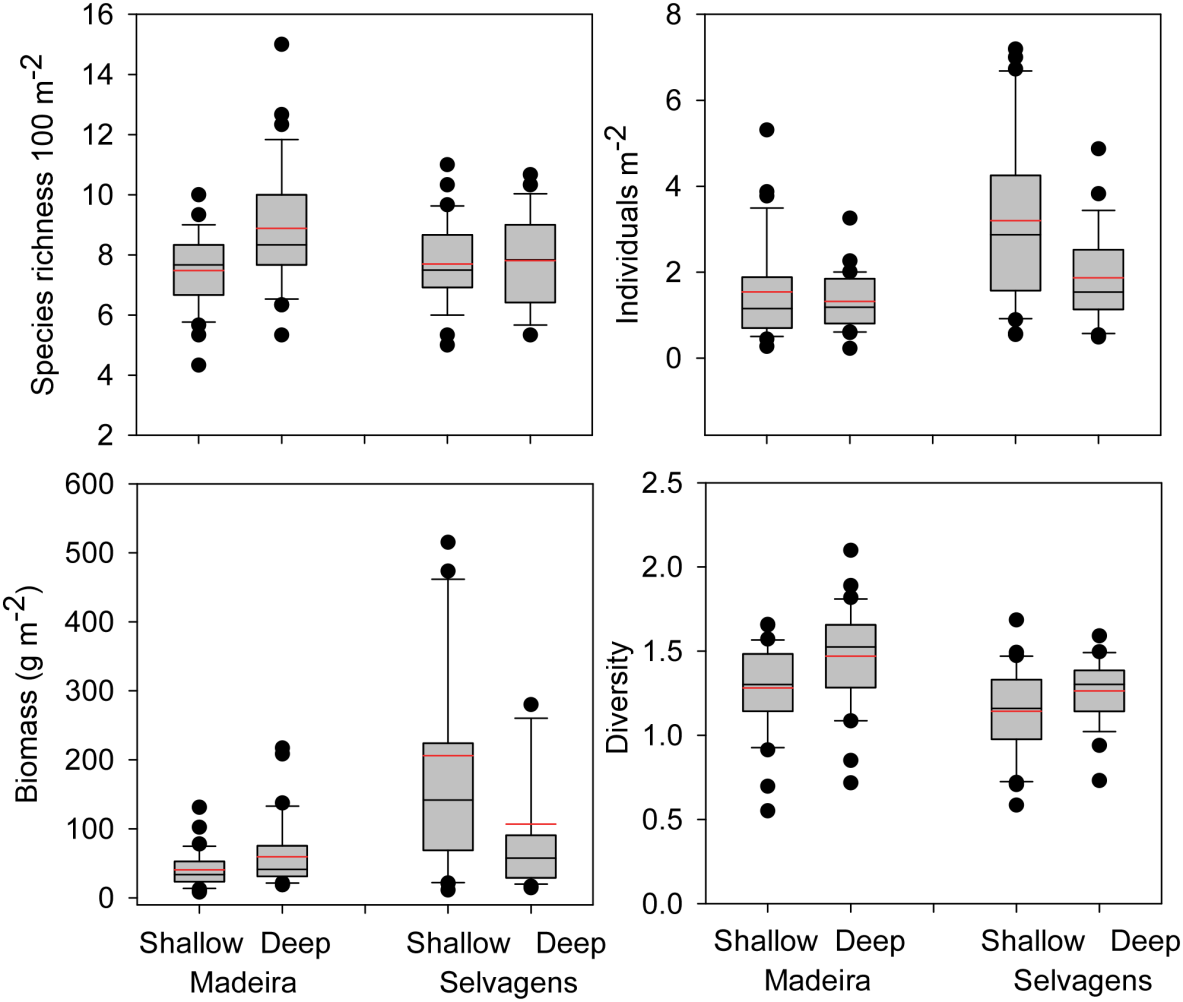
Comparisons of fish assemblage characteristics between islands and depths. Box plots showing median (black line), mean (red dashed line), upper and lower quartiles, and 5th and 95th percentiles. A. Species richness transect, B. Number of individuals m^-2^, C. Grams m^-2^, and D. Shannon-Weiner Diversity.

Overall species richness per transect was similar between islands, but there was a significant interaction between depth and island, with the 20 m sites at Madeira having significantly higher species richness compared with the other three depth x island combinations (Table 3, Fig 4). Density (number of individuals m^-2^) was significantly greater at Selvagens compared with Madeira, and significantly higher in the 10 vs. 20 m depth stratum. Total fish biomass (g m^-2^) was 3.2 times higher at Selvagens compared to Madeira, but there was a significant interaction between island and depth, with deep sites at Madeira not significantly different from deep sites at Selvagens despite an 80% higher biomass at the latter. Biomass of resource species followed a similar pattern. Diversity was higher at Madeira compared with Selvagens and higher in 20 m compared with 10 m.

**Table 3:** Comparisons of fish assemblage characteristics between islands and depth strata. Results of Linear Mixed Models with station as a random effect, and Tukey-Kramer HSD (honestly significant difference) pairwise comparisons among fixed factors. Underlined island x depth combinations are not significantly different (α = 0.05). Mad.–Madeira, Sel.–Selvagens.

Assemblage characteristic	Factor	F	P	Multiple comparisons
Species	Island	1.80	0.183	
	Depth	5.38	0.023	20 m > 10 m
	Is x depth	4.75	0.032	Mad. 20 m Sel. 20 m Sel. 10 m Mad. 10 m
Number	Island	21.72	<0.001	Selvagens > Madeira
	Depth	5.29	0.024	10 m > 20 m
	Is x depth	3.27	0.074	
Biomass	Island	26.66	<0.001	Selvagens > Madeira
	Depth	1.23	0.271	
	Is x depth	10.86	0.001	Sel. 10 m Sel. 20 m Mad. 20 m Mad. 10 m
Resource Biomass	Island	25.76	<0.001	Selvagens > Madeira
	Depth	1.68	0.199	
	Is x depth	11.85	<0.001	Sel. 10 m Sel. 20 m Mad. 20 m Mad. 10 m
Diversity	Island	14.58	0.002	Madeira > Selvagens
	Depth	11.39	0.001	20 m > 10 m
	Is x depth	0.61	0.435	

#### Fish assemblage structure

**Fig 5:**
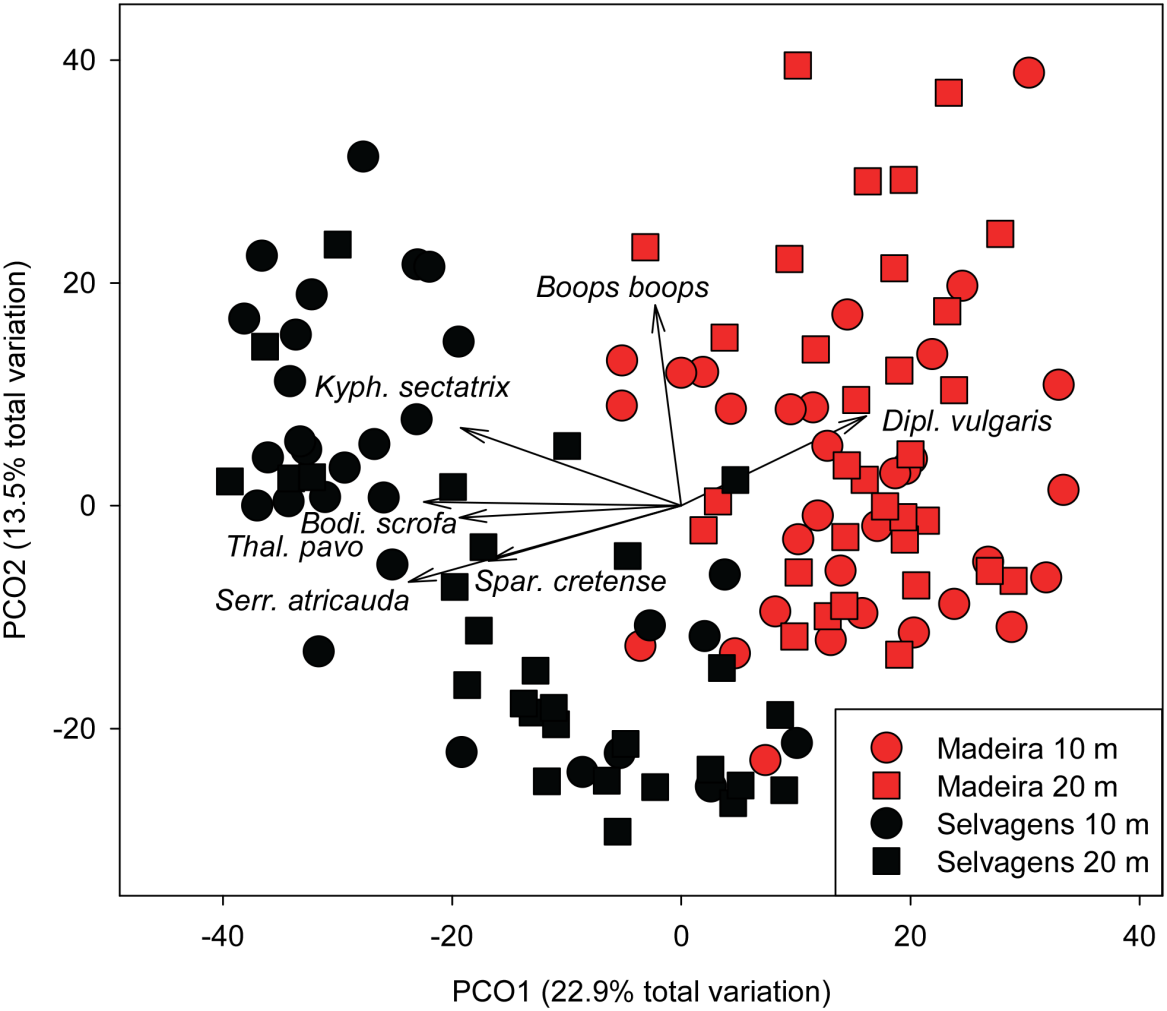
Principal Coordinates Analysis of fish species biomass. Square root transformation. Bray Curtis Similarity matrix.

Fish biomass assemblage structure was significantly different between Madeira and Selvagens (pseudo-F_1,121_ = 24.5, p < 0.001), between depths (pseudo-F_1,121_ = 4.4, p < 0.001), as well as their interaction (pseudo-F_1,121_ = 4.1, p < 0.001). Fish assemblage structure based on numerical abundance showed similar patterns with significant differences between Madeira and Selvagens (pseudo-F_1,121_ = 21.7, p < 0.001), between depths (pseudo-F_1,121_ = 5.9, p < 0.001), as well as their interaction (pseudo-F_1,121_ = 4.0, p = 0.004). Fish assemblage structure based on species biomass showed clear separation in ordination space between Selvagens and Madeira based on Principal Coordinates Analysis (Fig 5). The 1^st^ PCO axis explained ~23% of the variation in assemblage structure. This separation was driven by *Diplodus vulgaris* towards Madeira and *Kyphosus sectatrix*, *Serranus atricauda*, *Thalassoma pavo*, *Sparisoma cretense*, and *Bodianus scrofa* towards Selvagens. *Boops boops* was orthogonal to this primary axis. Average dissimilarity between islands was 79.2% and was driven by *K*. *sectatrix* (22.6%), *S*. *cretens*e (8.6%), *B*. *boops* (8.3%), and *T*. *pavo* (7.8%) (Table 4). Average dissimilarity between depth strata was 72.3% and was driven by *K*. *sectatrix* (17.7%), *B*. *boops* (10.8%), *Chromis limbata* (9.3%), and *S*. *cretens*e (7.4%) (Table 4).

**Table 4:** A. SIMPER for fish species by biomass (g m^-2^) most responsible for the percent dissimilarities between islands using Bray-Curtis similarity analysis of hierarchical agglomerative group average clustering. Average dissimilarity = 79.2%, with one standard deviation of the mean in parentheses. B. SIMPER for fish species by biomass most responsible for the percent dissimilarities between depths. Average dissimilarity = 72.3%. Abundance values are means and one standard deviation of the mean in parentheses.

**A**	**Species**	**Madeira**	**Selvagens**	**Avg Diss**.	**% contrib**.	**Cum. %**
**Kyphosidae**	*Kyphosus sectatrix*	0.3 (1.6)	54.2 (98.2)	17.9 (0.7)	22.6	22.6
**Scaridae**	*Sparisoma cretense*	4.8 (4.5)	12.5 (8.6)	6.8 (1.1)	8.6	31.2
**Sparidae**	*Boops boops*	7.3 (14.7)	6.1 (13.4)	6.6 (0.7)	8.3	39.5
**Labridae**	*Thalassoma pavo*	3.3 (4.6)	11.4 (8.4)	6.2 (1.2)	7.8	47.3
**Pomacentridae**	*Chromis limbata*	5.3 (6.3)	15.9 (76.4)	6.0 (0.6)	7.6	54.9
**Serranidae**	*Serranus atricauda*	0.9 (0.9)	5.6 (3.0)	3.6 (1.2)	4.6	59.4
**Sparidae**	*Sarpa salpa*	4.4 (13.5)	3.9 (115.8)	3.5 (0.5)	4.4	63.8
**Carangidae**	*Seriola dumerili*	-	24.9 (159.2)	3.2 (0.3)	4.1	67.9
**Labridae**	*Bodianus scrofa*	0.9 (1.8)	3.9 (4.4)	2.8 (0.8)	3.5	71.4
**Pomacentridae**	*Abudefduf luridus*	3.6 (3.9)	3.9 (2.4)	2.5 (0.8)	3.2	74.5
**B**	Species	Madeira	Selvagens	Avg Diss.	% contrib.	Cum. %
**Kyphosidae**	*Kyphosus sectatrix*	44.3 (95.6)	7.6 (28.3)	12.8 (5.6)	17.7	17.7
**Sparidae**	*Boops boops*	8.5 (16.3)	4.9 (11.3)	7.8 (0.7)	10.8	28.6
**Pomacentridae**	*Chromis limbata*	4.8 (7.7)	15.9 (74.3)	6.7 (0.7)	9.3	37.8
**Scaridae**	*Sparisoma cretense*	8.9 (8.3)	8.0 (7.3)	5.4 (1.0)	7.4	45.3
**Sparidae**	*Sarpa salpa*	4.4 (15.5)	4.0 (13.7)	4.3 (0.5)	5.9	51.2
**Labridae**	*Thalassoma pavo*	8.9 (9.1)	5.4 (5.8)	4.0 (0.9)	5.6	56.7
**Pomacentridae**	*Abudefduf luridus*	4.2 (3.5)	3.3 (3.0)	3.0 (0.7)	4.2	60.9
**Carangidae**	*Seriola dumerili*	20.6 (155.0)	3.1 (14.2)	2.4 (0.2)	3.3	64.1
**Sparidae**	*Diplodus sargus*	1.5 (5.6)	1.2 (3.0)	2.3 (0.4)	3.2	67.3
**Labridae**	*Bodianus scrofa*	1.7 (2.9)	2.9 (4.1)	2.3 (0.7)	3.1	70.4

There were large differences in the biomass of important resource fish species between Selvagens and Madeira (Table 4). Chubs (*K*. *sectatrix*) accounted for 34% of the biomass at Selvagens, with absolute biomass 182% higher than at Madeira. The amberjack *Seriola dumerili* was one of the most important species by weight at Selvagens (16% of total biomass), but was not observed on transects around Madeira. Another amberjack, *S*. *rivoliana*, was 7 times more abundant at Selvagens compared to Madeira. Barred hogfish (*B*. *scrofa*), a sea urchin predator, was 3.4 times more abundant by weight at Selvagens compared to Madeira. Another predator of small sea urchins, *S*. *cretense* [20], was 6.8 times more abundant by weight at Selvagens compared with Madeira. A total of fifteen Dusky Grouper (*Epinephelus marginatus*), which is listed as endangered by IUCN, were observed around Selvagens, ranging in size from 40 to 120 cm. Only four dusky groupers were observed around Madeira and these were all exclusively found within the Garajau MPA.

#### Fish trophic structure

**Fig 6:**
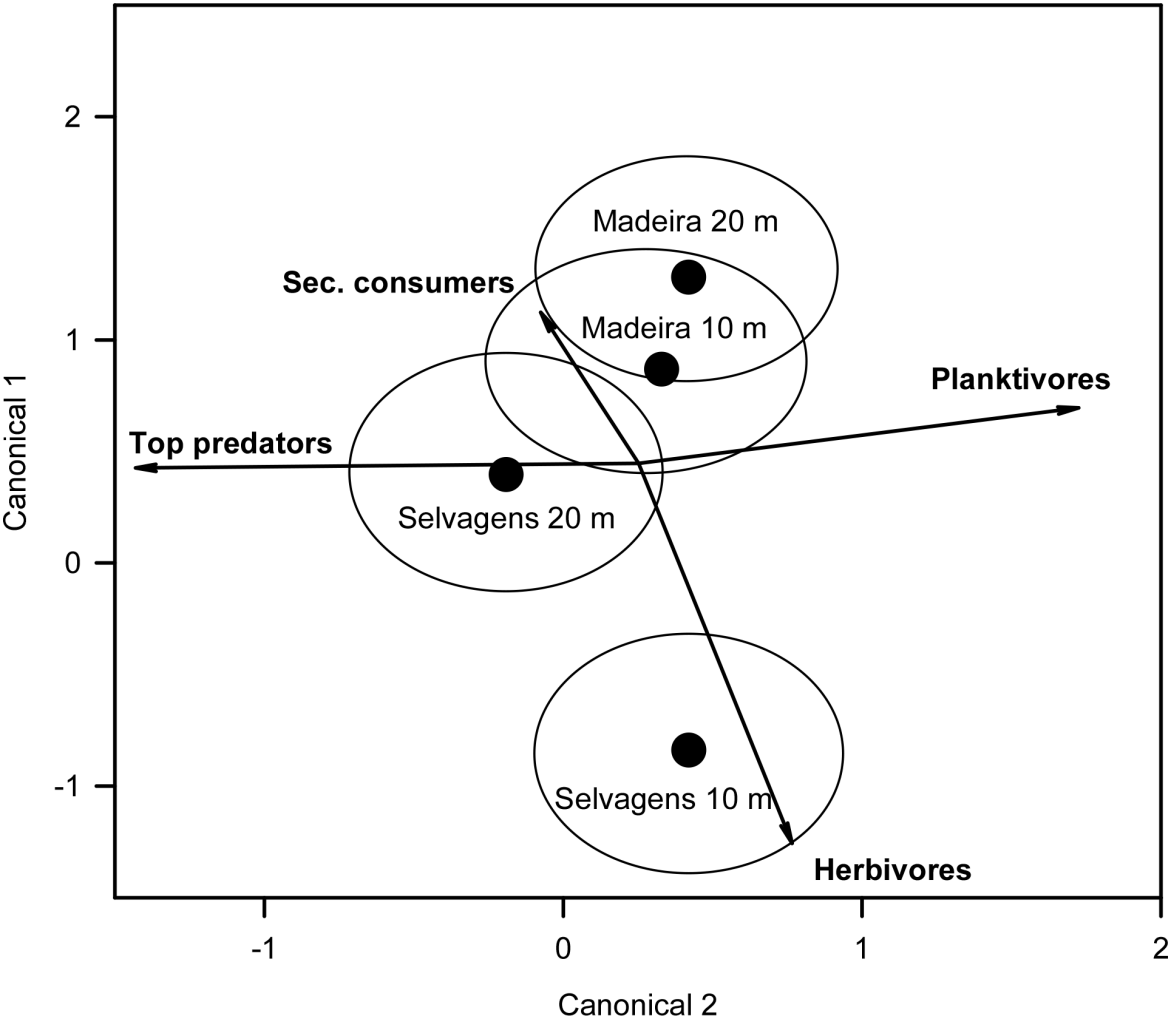
Canonical discriminate analysis of the difference in trophic group biomass between island groups and depths. Treatment (island group x depth) centroids and 95% confidence clouds are plotted together with the direction and importance of trends in trophic group.

Trophic structure based on biomass was significantly different between island groups (F_1,118_ = 26.85, p < 0.001) but not depth (F_1,118_ = 0.10, p = 0.756); however, the interaction between island and depth was significant (F_1,118_ = 5.56, p = 0.020). The 10-m stratum at Selvagens was distinct from the other three island x depth combinations, with herbivores most responsible for this separation (Fig 6). The Selvagens 20 m stratum was distinct from the 20 m Madeira stratum but overlapped with the Madeira 10 m stratum. Top predators accounted for this separation and was orthogonal to herbivores.

**Fig 7:**
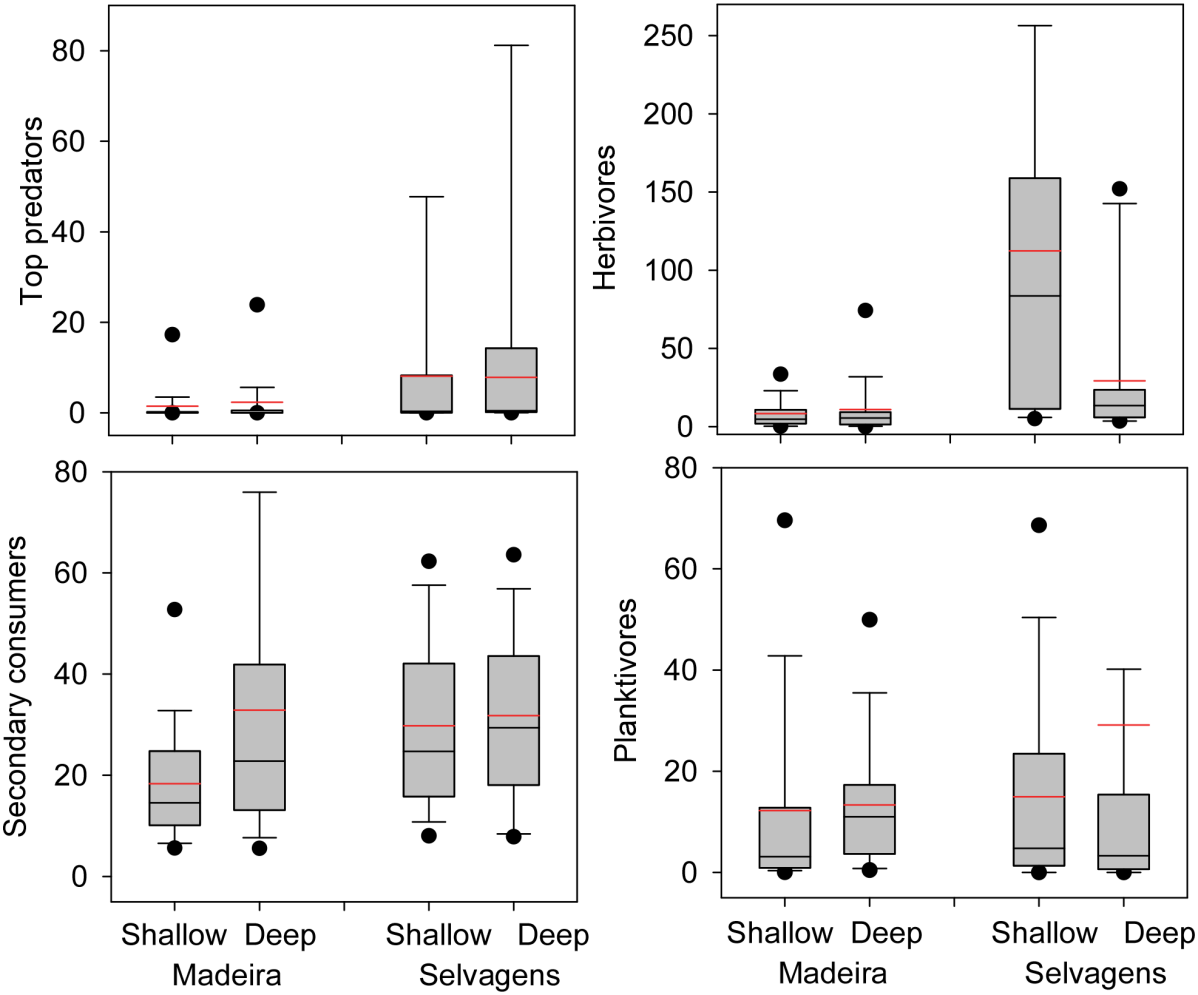
Comparisons of trophic biomass between islands and depths. Box plots showing median (black line), mean (red line), upper and lower quartiles, and 5^th^ and 95^th^ percentiles. A. Top predators, B. herbivores, C. secondary consumers, and D. planktivores. Values are in g m^-2^.

Biomass of top predators was an order of magnitude higher at Selvagens compared to Madeira, and accounted for 22% of total biomass at Selvagens but < 4% at Madeira (Table 5, Fig 7). Herbivore biomass was seven times higher at Selvagens compared with Madeira, but there was a significant interaction with depth. Herbivores comprised 45% of the total biomass at Selvagens and < 20% at Madeira. Secondary consumers had significantly higher biomass at Selvagens, as well as in the deep depth stratum. Planktivores were not significantly different between the two island groups or between depth strata.

**Table 5:** Comparisons of fish trophic biomass between islands and depth strata. Results of Linear Mixed Models with station as a random effect, and Tukey-Kramer HSD (honestly significant difference) pairwise comparisons among fixed factors. Underlined island x depth combinations are not significantly different (α = 0.05). Mad.–Madeira, Sel.–Selvagens.

Assemblage characteristic	Factor	F	P	Multiple comparisons
Top predators	Island	14.17	<0.001	Selvagens > Madeira
	Depth	0.20	0.654	
	Is x depth	0.01	0.972	
Herbivores	Island	62.81	<0.001	Selvagens > Madeira
	Depth	10.13	0.002	10 m > 20 m
	Is x depth	10.02	0.002	Sel. 10 m Sel. 20 m Mad. 20 m Mad. 10 m
Sec. consumers	Island	5.89	0.017	Selvagens > Madeira
	Depth	4.25	0.042	20 m > 10 m
	Is x depth	2.16	0.145	
Planktivores	Island	0.50	0.482	
	Depth	0.25	0.616	
	Is x depth	2.34	0.130	

#### Marine protected areas (MPAs)

**Fig 8:**
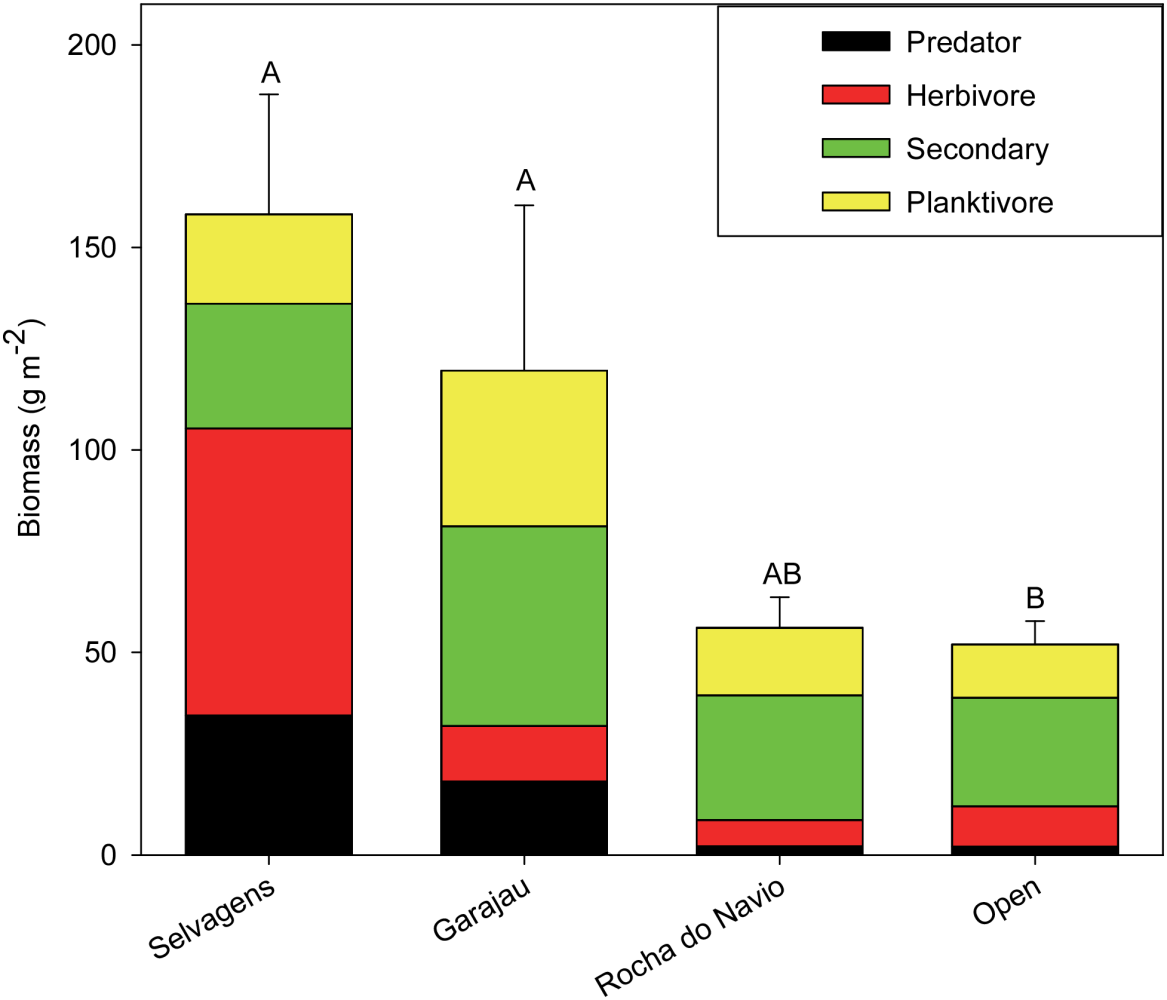
Comparisons of fish trophic biomass (g m^-2^) by management regime. Values are means and standard error of the mean. Kruskal-Wallis Rank Sum comparisons of total fish biomass among management regimes was statistically different (*X*^*2*^ = 46.3, p < 0.001). Management regimes with the same letter are not significantly different from one another (Dunn’s unplanned multiple comparisons procedures, α = 0.05).

Total fish biomass was significantly different among management regimes (*X*^*2*^ = 46.3, p < 0.001). Biomass in the Garajau MPA was 2.4 times higher than areas open to fishing around Madeira (p < 0.001) and 2.2 times higher than the partially protected Rocha do Navio MPA (Fig 8). The latter comparison, while not significant, is suggestive of differences between the two MPAs (p = 0.07). Total biomass at Garajau was only 1/3rd lower than Selvagens, while biomass at partially protected Rocha do Navio was 2.8 times lower than Selvagens. Top predators comprised 15% of the total biomass at Garajau but only < 4% at both Rocha do Navio and areas open to fishing around Madeira. Top predator biomass at Garajau was 90% lower than Selvagens, compared to Rocha do Navio, where top predator biomass was more than 15 times lower than Selvagens.

#### Whole community comparisons between islands

**Fig 9:**
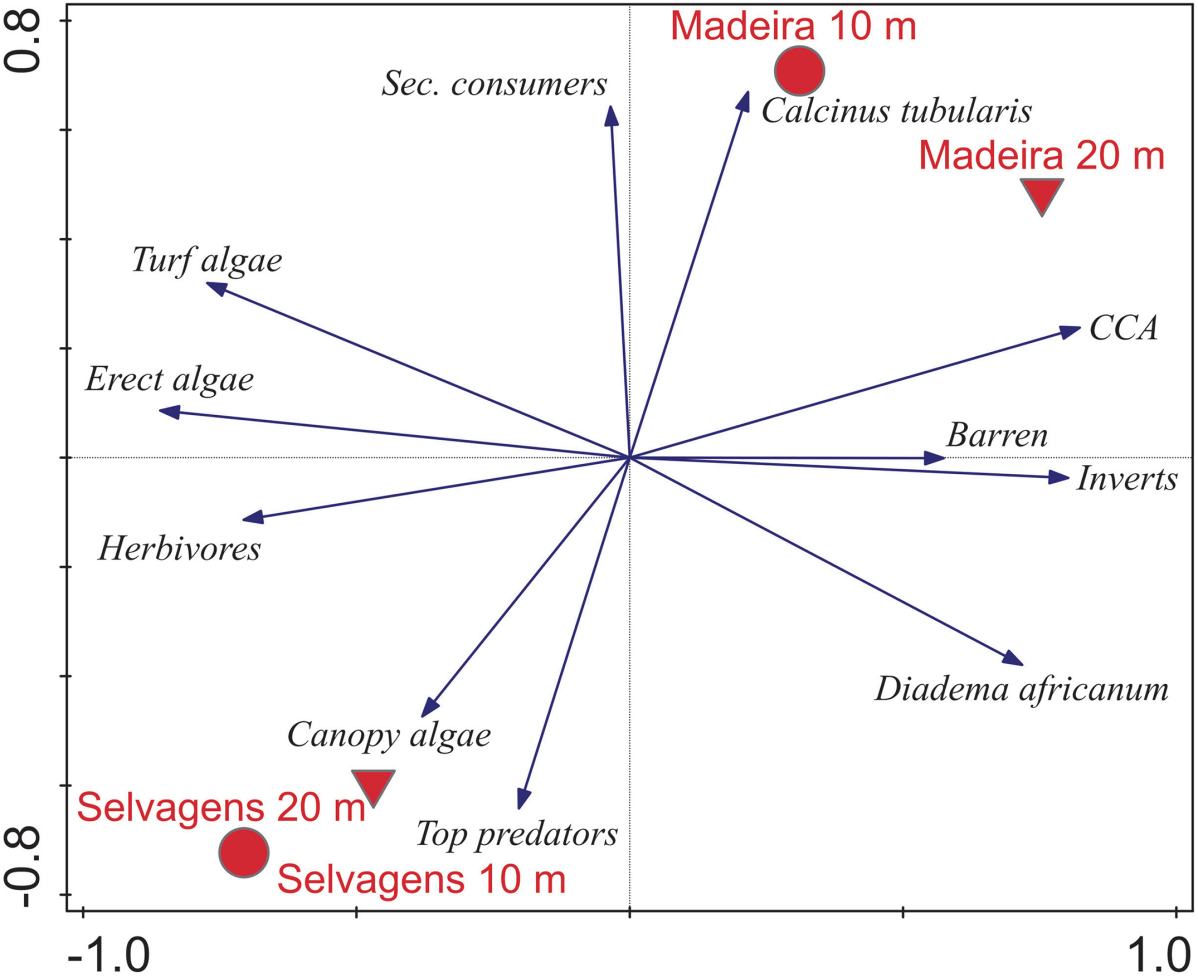
Biplot of results of Principal Components Analysis with supplemental variables on dominant sessile benthic cover, mobile invertebrates, and fish biomass by trophic group. Sessile benthic cover data were arcsine square root transformed, mobile invertebrates and fish biomass were ln(x+1) transformed prior to analysis. All data were centered and standardized. Statistical results are shown in Table 6.

There was clear separation between Madeira and Selvagens based on dominant sessile benthic cover, mobile invertebrates, and fish biomass by trophic group (Fig 9). Depth strata within islands were close to one another in ordination space, with the two depth strata at Selvagens having higher concordance compared to Madeira. The first two axes of the PCA biplot explained 46.5% of the variance in community structure and 63% of the community-island x depth relationships (Table 6). The major drivers of this separation for Selvagens were fish biomass of top predators and canopy algae. The separation for Madeira was driven by the hermit crab *C*. *tubularis*, secondary consumers and CCA. Erect algae, turf, and herbivore biomass were orthogonal to the primary vectors of island separation towards Selvagens, while barrens, invertebrates, and *D*. *africanum* were orthogonal in the direction of Madeira.

**Table 6:** A. Results of Principal Components Analysis with supplemental variables on dominant sessile benthic cover, mobile invertebrates, and fish biomass by trophic group.

A. Statistic	Axis 1	Axis 2	Axis 3
Eigenvalues	0.31	0.13	0.11
Explained variation (cumulative)	31.36	43.88	55.21
Pseudo-canonical correlation (suppl.)	0.55	0.48	0.59

## Discussion

The Selvagens and Madeira islands present a sharp contrast between a highly urbanized and developed island (Madeira) and a remote and virtually uninhabited marine reserve. The effects of fishing and land-based activities around Madeira have been well documented [31–34], and while we did not directly evaluate the anthropogenic stressors between Madeira and Selvagens, the extensive development and human activity at the former and the remoteness and near absence of human habitation at the latter clearly highlights the disparities in human pressure experienced between these two locations. As a result of these differences, the Selvagens Islands may represent one of the last remaining intact marine ecosystems in the eastern Atlantic.

Remote locations with limited fishing pressure and few land-based stressors are some of the few remaining examples of marine ecosystems without major anthropogenic influences [35–36]. Although the North Atlantic has been overexploited for centuries [37–39], the establishment of the Selvagens Islands Reserve in 1971 has resulted in the maintenance of a healthy ecosystem in a region where the surrounding seas are intensely fished and largely degraded. While illegal fishing has been reported around Selvagens, the presence of park rangers and the recent addition of a Portuguese maritime police force seems to have limited the impact of these activities as shown by the high abundance of large fisheries species, including the dusky groupers—one of the main targets of recreational and commercial fisheries in the nearby islands and in the Mediterranean.

**Fig 10:**
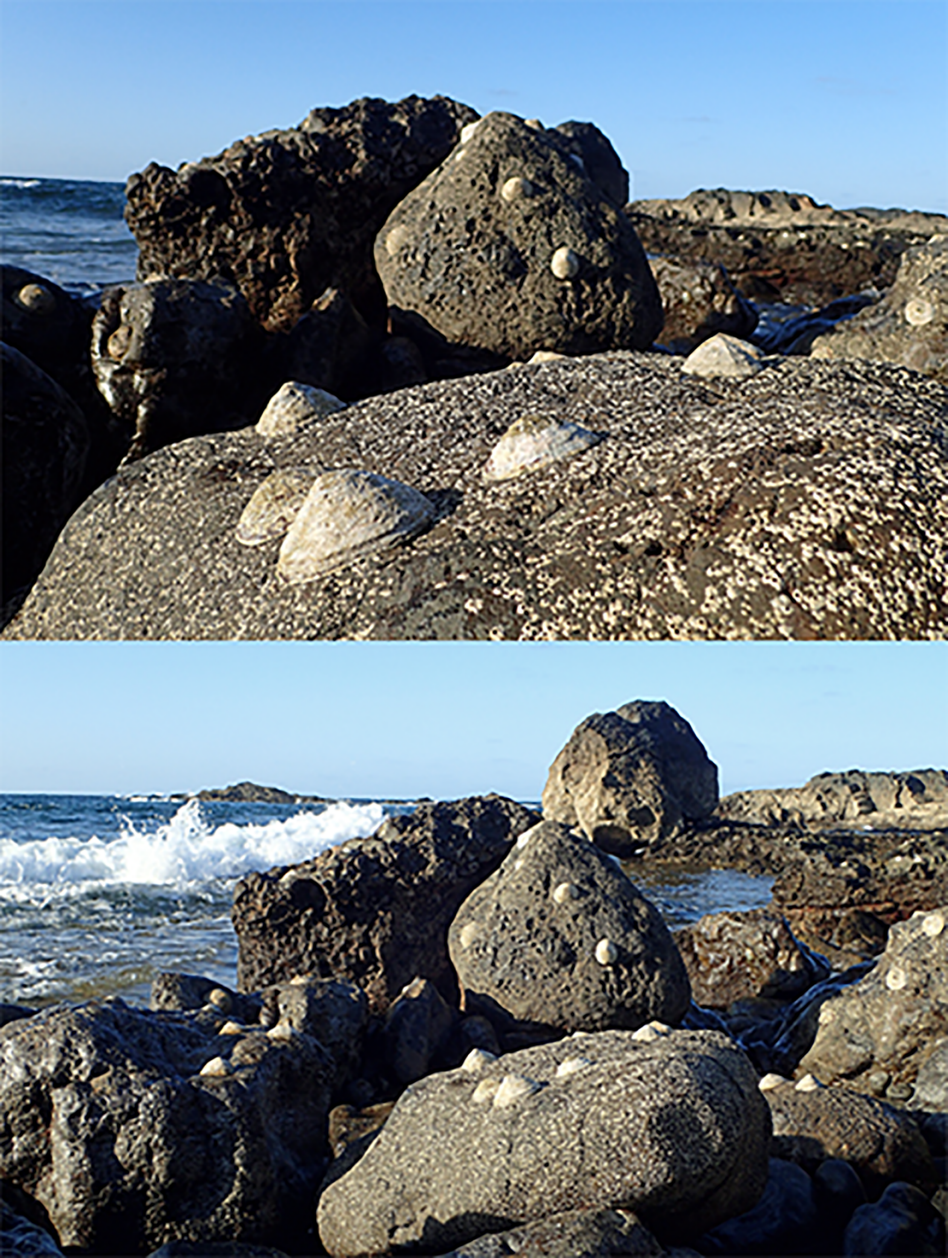
The intertidal habitats of Ilhas Selvagens are some of the least disturbed within the region, with the highly prized sun limpet (*Patella candei*) common and of large size, particularly around Selvagem Pequena.

The intertidal community around Selvagens was noteworthy for its abundance of large sun limpets (*Patella candei*), particularly on Selvagem Pequena (Fig 10). This species has become rare throughout much of its range due to overfishing and is listed as in danger of extinction in the Canary Islands [40]. In addition, top-shell snails (*Phorcus atratus*) and other limpets, mainly *Patella aspera* and *Siphonaria pectinata*, were also common. These extremely high densities of intertidal grazers appear to limit macroalgal growth, which was restricted to small patches of turf algae, mainly *Jania* cf. *rubens* in the lower intertidal. The intertidal around Selvagens likely represents one of the few remaining intact ecosystems of its kind in the region.

Turf algae and erect algae accounted for the majority of the sessile benthic cover around Selvagens, while Madeira was dominated by CCA, turf algae, sessile invertebrates, and sea urchin barrens. Our results from Madeira are consistent with previous studies of the benthos around this island [31–33]. Erect macroalgae provides the main biological substrate for many organisms in the eastern Atlantic [17, 41–42]. In the nearby Canary Islands, upright seaweeds are the principal engineering organisms on shallow rocky bottoms, providing complex habitats that support highly diverse communities [17, 43].

The sea urchin *D*. *africanum* was the most common mobile invertebrate observed at both islands, but densities were 65% higher at Madeira compared with Selvagens. The densities of *D*. *africanum* that we observed around Madeira are similar to those documented by researchers back in the 1990s and early 2000s [31–33]. In the eastern Atlantic, removal of top predators because of overfishing has been linked to hyperabundances of sea urchins (predominantly *Diadema*), with the subsequent elimination of erect vegetative frameworks and the creation of barrens as an alternate stable state [17, 20, 43–47]. These upright seaweed beds exist due to the balance between seaweeds, herbivores, and predators [19, 48], but human-derived stressors (e.g., overfishing, pollution) can reduce the resilience of these desirable macroalgal beds, while strengthening the influence of urchin barrens, and thus exacerbating the threat, spatial extent, and irreversibility of an unwanted regime shift [17–18, 21]. Previous studies around Madeira have shown similar negative relationships between sea urchin densities and macroalgal cover [32–33].

The identity of predatory fishes on the sea urchin *D*. *africanum* is well known from previous studies in the Canary Islands by directly assessing the frequency of predation events and indirectly through examination of fish stomach contents [20]. These authors also showed that the depletion of sea urchin predators in fished areas compared with MPAs resulted in increased sea urchin populations and cascading effects that reduces benthic diversity in areas open to fishing. Species known to feed on and control sea urchins (e.g., *Balistes capriscus*, *B*. *scrofa*, *Canthidermis sufflamen*, and *S*. *cretense*, [20]) were in high abundance at Selvagens compared to Madeira, where they are also important fisheries species.

Total fish biomass was more than three times higher at Selvagens compared to Madeira, and biomass of top predators was an order of magnitude greater. Several commercial species (e.g., groupers, jacks, triggerfishes) that have been overfished elsewhere in Macaronesia [16–18, 49], but were common and of large size at Selvagens compared with Madeira. Previous studies of the fish assemblages around Madeira dating back to the mid-1990s documented few large predatory fishes or other species with high resource value [24, 50–51], suggesting that these species may have been subjected to overfishing for many years.

The IUCN endangered Dusky Grouper (*E*. *marginatus*) is the best-known grouper of the Mediterranean Sea and adjacent European and North African coasts, but it has been overexploited throughout much of its range [52–53]. The prevalence and large sizes of this species at Selvagens is striking compared to Madeira, where it was only found within the highly restricted Garajau MPA. A similar pattern has been observed in other Macaronesian regions such as in the Canary Islands, where groupers are much more abundant around islands where fishing activities are restricted by MPAs and human population is low [16]. The Garajau MPA was established in 1986 and was the first exclusively marine protected area in Portugal. Overall biomass and that of top predators and secondary consumers was higher within this MPA when compared with areas open to fishing around Madeira and was comparable to Selvagens; however, its small size (3.8 km^2^) likely results in limited benefits beyond its boundaries.

The effects of fishing and other anthropogenic influences associated with urbanization are evident around Madeira [15, 34, 54–55]. This is in stark contrast to Selvagens, which harbors rich benthic communities with diverse algal assemblages and high fish biomass, along with an abundance of large resource species. The clear differences between these two islands highlights the importance of protecting the Selvagens, which harbors one of the last intact marine ecosystems in the North Atlantic, and the need to increase management and protection around Madeira if the ecosystem is to recover and provide the ecosystem services essential to the island community. No-take areas have been identified as an effective tool to restore erect macroalgal beds in other Macaronesian islands [56], and are known to provide overall ecosystem benefits both within and beyond their borders [57].

Benthic and fish communities at Selvagens resemble those of some MPAs around other areas of Macaronesia. Densities of the most common mobile invertebrate *D*. *africanum* at Selvagens are within the range recorded in the no-take reserve of La Graciosa in the Canary Islands [45]. However, sea urchin abundances are still an order of magnitude higher than densities recorded at the most effective MPA in the Canary Islands, Mar de Las Calmas, on El Hierro Island [45]. Similarly, fish biomass at Selvagens were similar to those in the La Graciosa no-take reserve, but lower than those in the Mar de Las Calmas [58]. These differences may be driven by inherent characteristics of both Selvagens and the Canary Islands, with the former showing a higher proportion of warm-temperate species and the latest having more complex and variable assemblages, likely due to the larger sizes of the islands and more heterogeneous habitats [59].

Threats to the Selvagens include illegal fishing within the reserve and unregulated or weakly monitored fishing for tuna and other target species surrounding the reserve. The current 200 m depth limit for the reserve means that fishing can occur very close to the islands, with potential impacts to nearshore species. Expansion of the reserve would provide protection for coastal and pelagic species, as well as reducing by-catch of sea birds, marine mammals, and large pelagic fishes (e.g., tuna and billfishes) that frequent the area. In addition, the Selvagens may be an important biogeographical link between Madeira and the Canary Islands [60].

The results of this study reinforce patterns observed at smaller spatial scales within the Canary Islands [16–17, 45–46], and are consistent with observations contrasting remote vs. inhabited marine ecosystems around the world [61–63]. The Selvagens harbor one of the last intact marine ecosystems in the North Atlantic, and maintains high coastal fish species diversity within a relatively small area [60]. The Selvagens Nature Reserve serves as a global model for what can be achieved elsewhere if species are protected and allowed to recover within their borders. Increased protection for this unique area is a precautionary bulwark against the degradation and decline of marine ecosystems throughout the region. Our results also identify the need for better fisheries and coastal zone management, as well as the need for larger and more effective marine protected areas around Madeira if the ecosystem is to recover and provide the ecosystem services essential to the island community.
